# Razor scrape assay, an alternative variation to wound and healing assays

**DOI:** 10.1016/j.mex.2020.101135

**Published:** 2020-11-10

**Authors:** Jesús Antonio Lara-Reyes, Mariana Giezi Jimenez-Buendia, Gonzalo Emiliano Aranda-Abreu, Deissy Herrera-Covarrubias, Clara Luz Sampieri, Arnoldo Aquino-Galvez, Jorge Manzo-Denes, María Elena Hernández-Aguilar, Fausto Rojas-Durán

**Affiliations:** a Doctorado en Investigaciones Cerebrales, Universidad Veracruzana, Xalapa, Veracruz, México; b Centro de Investigaciones Cerebrales, Universidad Veracruzana, Xalapa, Veracruz, México; c Instituto de Salud Pública, Universidad Veracruzana, Xalapa, Veracruz, México; d Instituto Nacional de Enfermedades Respiratorias, Ciudad de México, México

**Keywords:** Cell culture techniques, Cell migration assays, Cell movement

## Abstract

Cell migration is the process by which cells move through tissues, and it is crucial to carry out a wide variety of physiological and pathological processes. The study methods to evaluate cell migration are very useful tools for biomedical research. Among these methods, the wound and healing assay is one of the simplest, most economical and is widely used in research. However, one of its disadvantages is that the width and shape of the wound can vary among experimental samples since the scraping is carried out manually, representing a difficult variable to control. In the present article a variant of the razor scrape assay is addressed, which eliminates this variation in the width of the wound, thus facilitating the measurement and comparison using the total area of cell migration.

• A method that can be carried out under standard culture conditions.
• Avoids the disadvantage of variation in width and shape of the wound.
• It constitutes a simple, cheap option and multiple advantages over the traditional method.

## Graphical abstract

**Figure:**
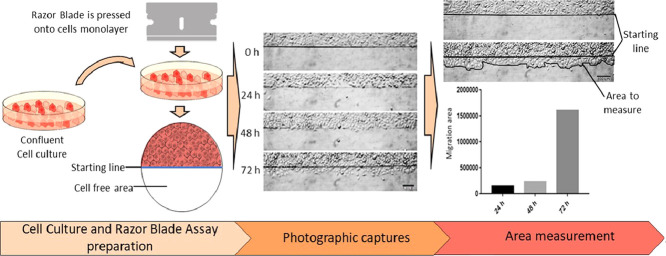

**Table:** Specifications Table

Subject Area	Biochemistry, Genetics and Molecular Biology
More specific subject area	Cell Biology of Cell Migration
Method name	Razor scrape assay
Name and reference of original method	Simoncini T, Scorticati C, Mannella P, Fadiel A, Giretti MS, Fu X-D, et al. Estrogen Receptor α Interacts with Gα _13_ to Drive Actin Remodeling and Endothelial Cell Migration via the RhoA/Rho Kinase/Moesin Pathway. Mol Endocrinol 2006;20:1756–71. Da Silva PL, Do Amaral VC, Gabrielli V, Montt MM, Mannella P, Baracat EC, et al. Prolactin promotes breast cancer cell migration through actin cytoskeleton remodeling. Front Endocrinol (Lausanne) 2015;6:1–8.
Resource availability	ImageJ: https://imagej.nih.gov/ij/download.html

Cell migration is the process by which cells move through tissues or on the surface of a culture plate (in vitro). It is crucial to carry out a wide variety of developmental and physiological processes, such as; embryo development, immune response, wound healing, etc., as well as in many pathological processes like inflammation and metastasis in cancer [1]. Because increased migration is the main process for cancer cells to invade and metastasize [2], knowing the mechanisms that control cell movement is one of the main goals of the research [3].

Study methods to evaluate cell migration are very useful tools for various disciplines in the biomedical sciences. Among the different methods available, the wound and healing assay is one of the simplest and cheapest. It provides information on the ability of cells to migrate collectively or individually, and morphological characteristics can even be observed during migration [1]. By measuring the distance or area of wound closure at a certain time, compared to a control, specific changes in migration can be revealed, including differences in the formation of lamellipodia, filopodia and direction movement.

Due to its importance, a great variety of techniques and commercial kits have been developed for its study. Several wound and healing assays based on mechanical, chemical, optical and electrical methods have been developed to create monolayer wound models with cells to study the process of cell migration. These approaches can result in different microenvironments for cells and possess diverse characteristics in terms of complexity, development, reproducibility and flexibility. However, low cost alternatives to carry out wound and healing studies are very few.

The wound and healing assay is widely used in research as it can be carried out under standard culture conditions using equipment available in most cell culture laboratories [4]. It is based on the observation of the behavior of a monolayer of confluent cells on a culture surface in which a wound or gap has been made with a micropipette tip, which will initially be a cell free area, the cells will move towards the inside area of it, images are captured in certain periods of time and compared to determine the differences.

One of the greatest advantages of this method is that no specialized material is required for the assay, which means they can be done in common cell culture multi-well plates and with a microscope with low magnification objectives (4X and 10X) regularly used in cell culture. Additionally, different types of cell matrices can be used if required.

However, this assay has some disadvantages, such as; variations in cell confluence; damage to the extracellular matrix layer as it is done through a scraping mechanism as well as variations in the width and shape of the wound between wells of the same experiment since they are carried out manually, making it difficult to measure and compare them [5].

Regarding this last point, a razor scrape assay has been described, which eliminates this variation in the width of the wound, facilitating measurement and comparison. In this variant a razor is pressed on the monolayer of cells in the plastic culture dish, thereby marking the starting line. The cells are removed on one side of that line and photographed at the beginning and at certain periods of time, the distance of the migration is measured in micrometers taking the starting line as a reference [6,7].

In this article we described a protocol base on this method modifying the measurement procedure, changing the distance per area. This method was applied in our studies with the MCF-7 breast cancer cell line. We hope that the description of this low-cost method will be useful in the investigation of cell migration.

## Method details

### Materials

**Table:** 

Materials and reagents	e.g. company	Comments
MCF-7 cell line	ATCC	This cell line was used for illustrative purposes only, any adherent cell line can be used in this method.
90 mm dishes	Corning	Routine cell culture and propagation.
60 mm dishes	Corning	Used for razor assay
RPMI-1640 culture medium	LONZA	Any other medium can be used.
Fetal bovine serum (FBS)	Biowest	Any other can be used.
Penicillin/streptomycin	Sigma/Aldrich	Any other can be used.
CO_2_ incubator	Nuaire	Any other can be used.
Razor blades		Single (recommended) or double edge

### Cell culture

Culture the cells to be used in the appropriate medium and supplementation until you have enough cells to do a passage. In the present method the human breast cancer cell line MCF-7 was routinely cultured in sterile 90 × 20 mm Petri dishes (46 cm^2^ growth area) (Corning), using RPMI-1640 culture medium without L-glutamine (LONZA) supplemented with non-inactivated FBS(Biowest) 8%%(v/v) and penicillin/streptomycin (Sigma/Aldrich) 1% (v/v) concentration. The cells were incubated using an incubator (Nuaire) maintained at 37°C and 5% CO_2_ atmosphere. The culture medium was changed every other day.

### Procedure

(1) Seed and culture cells in 60 mm culture dishes (21 cm^2^ growth area) under appropriate medium, supplementation and incubation conditions (usually 37 °C and 5% CO_2_ atmosphere) until they reach confluence >90%, that is, the percentage of the growing area surface that is covered with cells must be more than the 90%.

Note: the number of cells to be seeded and the time required to reach the confluence must be determined, which will depend on the size of the culture surface.

**Fig. 1:**
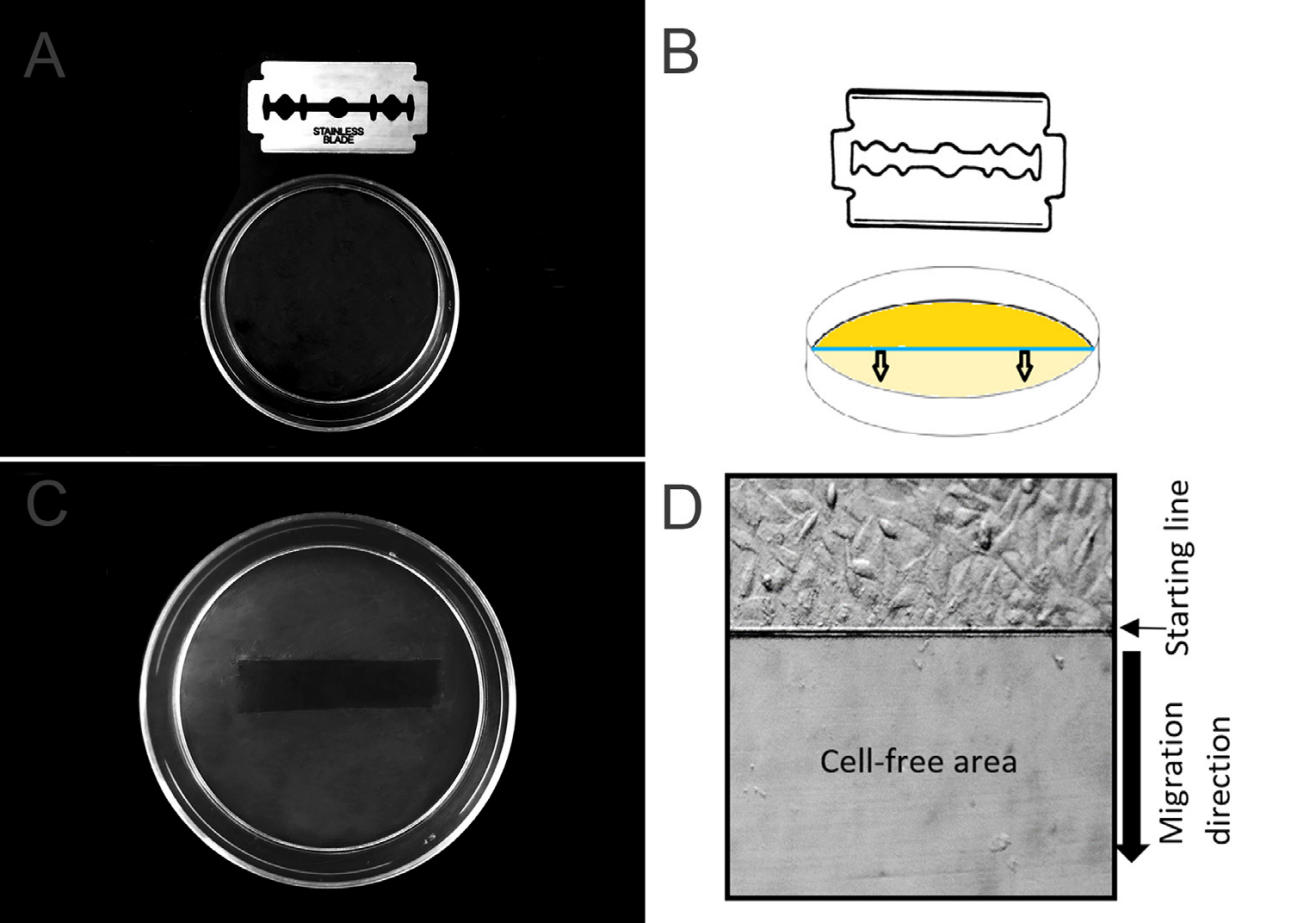
Cell migration assay, Razor scrape variation. (**A)** Plastic culture dish. (**B)** Razor blade and plastic culture dish. (**C)** Culture dish with cells monolayer and cell free area created by the razor blade. (**D)** Initial picture with the starting line and the cell free area (10X).
(3) All cells on one side of the marked line are removed. They can be swept away with a scraper or with the same razor blade, but care must be taken not to use excessive force to avoid damaging the plate surface (Fig. 1 C and D).
(4) Aspirate the culture medium and cell dendrites.
(5) Do 3 washes by slowly adding PBS to the well walls until the surface is completely covered and removed by aspiration.
(6) Slowly add culture medium with the corresponding substance to test along the walls of the well until completely covering the surface, taking care not to detach additional cells.

Note: It is recommended to perform the test under conditions of low serum concentration, which allows cells to migrate and does not cause cell death, but minimizes cell proliferation, since this could hide cell migration.

(2) In a biosafety cabinet (sterile environment) a sterile razor blade is pressed onto the cell monolayer on the plastic culture dish, thereby marking the starting line (Fig. 1 A and B).

(7) Observe under an inverted microscope, choose and mark the areas to be photographed.
(8) Take the initial photograph (Fig. 1 D).
(9) Put the culture plate in an incubator with the appropriate temperature and CO_2_ concentration (generally 37 °C and 5% CO_2_).
(10) Every certain period, remove the culture plate from the incubator, place it in the inverted microscope and take a photograph in the previously chosen and marked areas. In the Fig. 3 A, B and C we show an example of the MCF-7 cell migration analysis in different period of hours, only supplemented medium was used, without any different stimulus.

### Image analyses

**Fig. 2:**
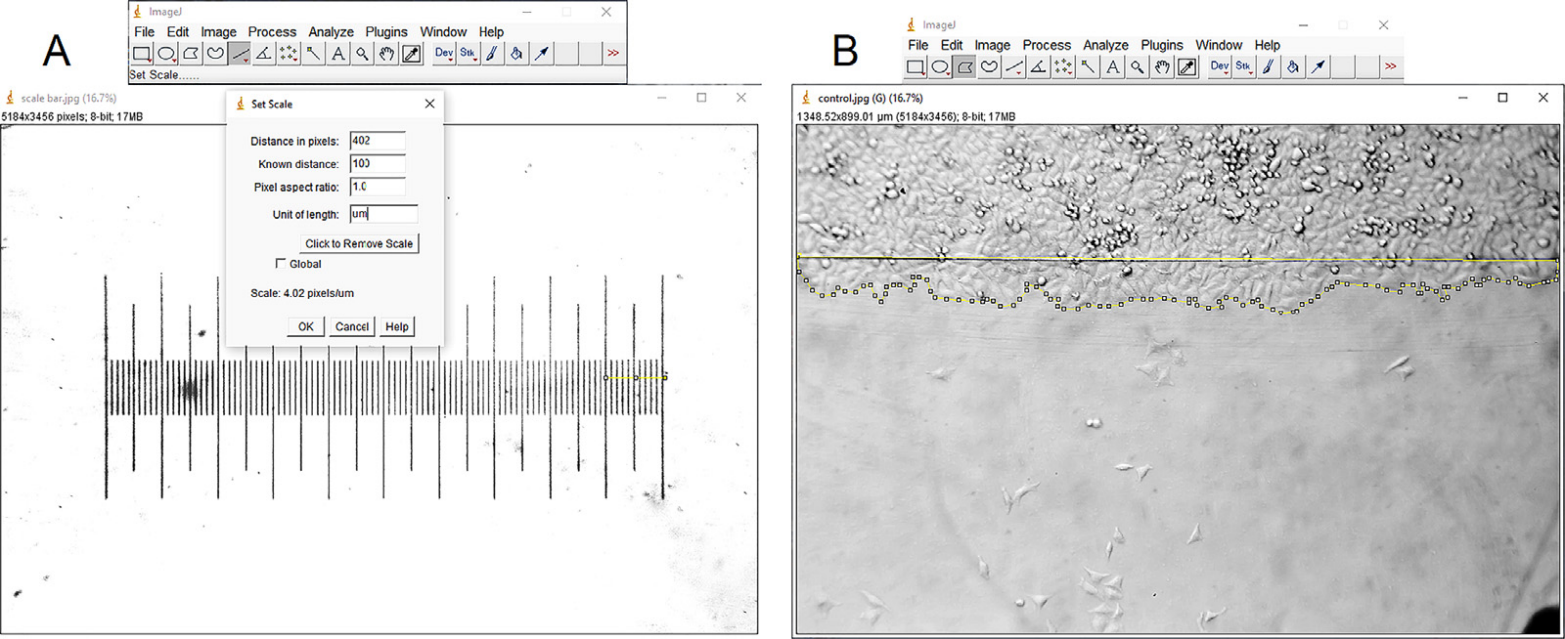
(A) Scale bar. (B) Selection of area to be measured.

To analyze the results in the captured images, the area covered by the cells will be measured taking the starting line as a reference (Fig. 1 D). The measurement in this case was carried out with the ImageJ program [8]. A picture of a scale bar is required to use as reference.

(A) Open the image file of the scale bar in ImageJ by selecting File ➔ Open ➔ select the image.
(B) Once the image is opened, it must be converted to 8 bits by selecting Image ➔ Type ➔ 8-bit.
(C) To assign the known distance from the scale bar using the Straight tool and selecting the known distance, in the present case 100 µm, then select Analyze ➔ Set scale ➔ introduce the known distance and units ➔ OK (Fig. 2 A).
(D) Without closing the scale bar image, open the image file of the cells (the same procedure described in step (A)) and convert the image to 8 bits (step (B)).
(E) Select the area occupied by the cell after the starting line using the Polygon selections tool, and then select Analyze ➔ Measure, a new window will open with the results of the measurement, in this case the unit will be µm^2^(Fig. 2 B).

**Fig.3:**
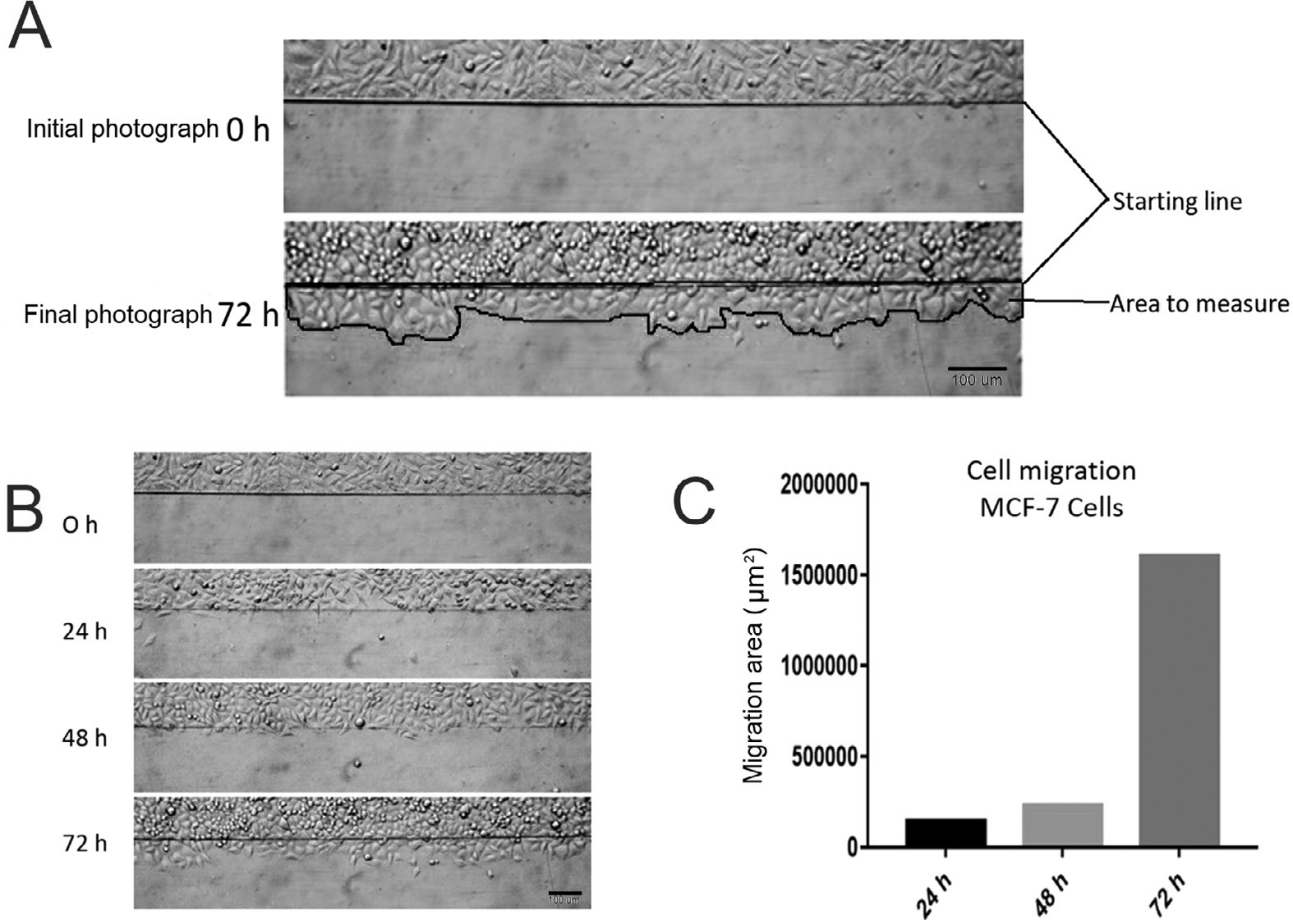
(A) Measurement of the migration. (B) and (C) Comparison between different periods of time. (B) the migration area at 24, 48 and 72 h and (C) the graphic representation of the migration area in μm^2^ are observed.

In this article we show a simple, and low-cost method, that allows to measure the total cell migration area. Choosing a wound and healing assay, as well as the appropriate method of analyzing the data, can be challenging the first time that cell migration research is approached. A good start would be to know what equipment is available. The wound and healing assay is a method that can be carried out under standard culture conditions and without equipment other than that in common use.

The adaptation of the assay described here was developed due to the difficulties encountered in measuring migration areas, it is simple to implement, does not require different material and equipment, and avoids the disadvantage of variation in width and shape of the wound, since by marking only one starting line, this constitutes a reference point from which measurements can be carried out in a simpler and more practical way. Likewise, the acquisition of images is relatively simple and only requires an inverted microscope that has an image capture system or allows the adaptation of a photographic camera. To analyze the data, the only thing needed is a program that allows the measurements to be made, such as the freely available ImageJ software. Therefore, it constitutes a simple, cheap option and multiple advantages over the traditional method.

## Declaration of Competing Interest

The authors declare that they have no known competing financial interests or personal relationships that could have appeared to influence the work reported in this paper.
